# Using a Discourse Task to Explore Semantic Ability in Persons With Cognitive Impairment

**DOI:** 10.3389/fnagi.2020.607449

**Published:** 2021-01-18

**Authors:** Malin Antonsson, Kristina Lundholm Fors, Marie Eckerström, Dimitrios Kokkinakis

**Affiliations:** ^1^Department of Swedish, Faculty of Arts, University of Gothenburg, Gothenburg, Sweden; ^2^Speech and Language Pathology Unit, Institute of Neuroscience and Physiology, Sahlgrenska Academy, University of Gothenburg, Gothenburg, Sweden; ^3^Institute of Neuroscience and Physiology, University of Gothenburg, Sahlgrenska Academy, Gothenburg, Sweden; ^4^Centre for Ageing and Health –AgeCap, University of Gothenburg, Gothenburg, Sweden

**Keywords:** discourse, mild cognitive impairment, language and aging, machine learning, semantic impairment

## Abstract

This paper uses a discourse task to explore aspects of semantic production in persons with various degree of cognitive impairment and healthy controls. The purpose of the study was to test if an in-depth semantic analysis of a cognitive-linguistic challenging discourse task could differentiate persons with a cognitive decline from those with a stable cognitive impairment. Both quantitative measures of semantic ability, using tests of oral lexical retrieval, and qualitative analysis of a narrative were used to detect semantic difficulties. Besides group comparisons a classification experiment was performed to investigate if the discourse features could be used to improve classification of the participants who had a stable cognitive impairment from those who had cognitively declined. In sum, both types of assessment methods captured difficulties between the groups, but tests of oral lexical retrieval most successfully differentiated between the cognitively stable and the cognitively declined group. Discourse features improved classification accuracy and the best combination of features discriminated between participants with a stable cognitive impairment and those who had cognitively declined with an area under the curve (AUC) of 0.93.

## Introduction

Dementia disorders are neurodegenerative diseases that affect millions of people each year, and the prevalence is still increasing (Scheltens et al., 2016). The most common type of dementia is Alzheimer's disease (AD), and despite extensive ongoing research, little is known about the cause. The development of most dementia disorders is gradual, and cognitive changes are detectable years, and sometimes decades, before dementia is diagnosed (Reisberg and Gauthier, 2008; Ritchie et al., 2015). Subjective cognitive impairment (SCI) and mild cognitive impairment (MCI) are two conditions that have been identified as states preceding dementia (Reisberg and Gauthier, 2008). MCI is characterized as a condition where cognitive decline is observable in at least one cognitive domain, but which does not have a significant interference with a person's daily life (Gauthier et al., 2006). In SCI, which is a common condition in the aging population and is characterized by mild cognitive complaints, no objectively observable cognitive decline is seen (Mendonça et al., 2016). However, previous longitudinal studies report that up to 44% of persons fulfilling the criteria for MCI may return to normal within a year (Gauthier et al., 2006). It is of clinical importance to identify which persons are at risk of cognitive decline and which are likely to remain cognitively stable. If differences in clinical profiles exist between the groups, this could be of help for clinicians diagnosing and planning the care for these groups. At present, there is no gold standard regarding what tasks to use to evaluate language function in persons at risk of developing dementia. However, what is known is that language deficits in general and specifically semantic difficulties are seen early on McCullough et al. (2019), and multiple evaluation methods might be needed to assess changes in language ability (Taler et al., 2020).

In this study we use a discourse task to explore aspects of semantic production in persons with various degrees of cognitive impairment and healthy controls. The purpose is to test if a semantic analysis of a cognitively and linguistically challenging discourse task can be used to differentiate persons with a progressive cognitive decline from those with a stable cognitive impairment. Both quantitative and qualitative measures of semantic ability are used for the purpose of answering this question.

## Background

Subtle changes in a person's speech or language use may be an early sign of cognitive decline. When a more pronounced cognitive decline, such as dementia, has developed, alterations in syntax, semantics and pragmatics are often present, whereas in milder forms of cognitive decline such as in MCI, predominantly semantic difficulties are seen (see e.g., Taler and Phillips, 2008). Recent studies have also found discourse related features to differentiate between persons with early cognitive impairments and healthy ageing, such as differences in cohesion (Kim et al., 2019) and global coherence (Mazzon et al., 2019). Substantial efforts have been made to identify markers that can be used to predict cognitive decline and that are associated with dementia. Since language data is relatively easy to collect compared to e.g., blood samples and brain imaging, many studies have focused on finding linguistic signs of early cognitive impairment using both qualitative and quantitative measures (for a review see e.g., Mueller et al., 2016) and exploring data both from language tests and continuous speech.

### Tests of Semantic Ability

In semantic verbal fluency (SVF) tasks a person is asked to produce as many items as one can from a certain category during 60 s. Although test of verbal fluency tests measure a combination of various cognitive functions and are commonly used to assess both verbal ability and executive control (Shao et al., 2014). SVF are often used for investigating semantic processing and production. Persons with MCI perform worse than healthy controls on SVF tasks, and research suggests that semantic retrieval is impaired (Demetriou and Holtzer, 2017; Linz et al., 2019). A decline in verbal fluency can in fact be seen very early as shown in a series of studies investigating late middle-aged individuals at risk for MCI, where those having “early” MCI had deficits in verbal fluency (Mueller et al., 2015, 2016; Johnson et al., 2018). Furthermore, a decline in semantic fluency in participants at the pre-MCI stage have been seen to predict later progression to MCI and dementia (Loewenstein et al., 2012). Another aspect of semantic ability is confrontation naming, often measured using the Boston Naming Test (BNT; Kaplan and Weintraub, 1983), which consists of 60 images in decreasing order of word frequency. In a recent meta-analysis, Belleville et al. (2017) assessed the predictive accuracy of different cognitive domains and found that in the language domain, confrontational naming (Ahmed et al., 2008; Eckerström et al., 2013) and SVF (Ahmed et al., 2008; Gallagher et al., 2010; Venneri et al., 2011) both yielded high predictive accuracy. Furthermore, numerous studies have shown a relationship between poor baseline performance on semantic word fluency and later development of dementia (Saxton et al., 2004; Auriacombe et al., 2006; Clark et al., 2009). Naming tests are widely used both clinically and in research and have been found to predict the speed of cognitive decline in AD (Carswell, 1999). However, the diagnostic and prognostic utility of these tests may be limited compared to other neuropsychological tests (Taler and Phillips, 2008), and they may not reflect actual ability to communicate and take active part in conversations (Reppermund et al., 2011). Nevertheless, naming tests have been found to correlate with lexical retrieval of nouns in connected speech for persons with aphasia (Herbert et al., 2008).

### Quantitative and Qualitative Analyses of Semantic Ability in Discourse

Whereas, quantitative ways of assessing language, such as language tests, have the benefit of being easy to administer and score, analysis of continuous speech, i.e., discourse, is assumed to have a higher sensitivity for detecting subtle linguistic impairments. Analysis of discourse not only allows for a detailed analysis of lexical, semantic, syntactic, and pragmatic features, but also for an analysis of temporal patterns of language production. In previous research, disfluencies (such as pauses, fillers, and false starts) have been studied as a proxy of word finding difficulties, i.e., semantic impairment. In a review, Boschi et al. (2017) conclude that speech in persons with AD is characterized by low speech rate and numerous hesitations. Further, Gayraud et al. (2011) showed that silent pauses, lengthenings, and hesitations are more common in the speech of persons with AD, but there is no increase in filled pauses, which can be interpreted as a lack of signaling speech production difficulties. While pauses may be seen as a symptom of semantic and lexical impairments, Pistono et al. (2019) suggest that pauses may indicate different types of difficulties, as they found that pauses in persons with AD appeared to be predicted by different cognitive functions, depending on the task, and the function of pauses may change as AD progresses (Davis and Maclagan, 2009). In that sense it should be noted that disfluencies are not solely indicative of word finding difficulties: individual differences may be related to verbal intelligence and working memory for example (Engelhardt et al., 2019). Persons with MCI tend to produce longer hesitations (Szatloczki et al., 2015), more pauses (Meilán et al., 2020) and have a lower speech rate (Szatloczki et al., 2015; Meilán et al., 2020). Although it is often concluded that disfluencies are early signs of cognitive decline, Mueller et al. (2016) found no difference in disfluencies between participants judged as having preclinical (early) MCI and participants who were cognitively healthy. However, in a more recent study by the same group involving more participants they could see that disfluencies in spoken discourse predicted early MCI status and that those with early MCI declined faster in measures of speech fluency than participants who were cognitively stable (Mueller et al., 2018).

Discourse is affected by semantic impairments, and researchers have investigated how aspects of spoken or written discourse are related to cognitive decline. A seminal study in the field, the Nun study (Snowdon et al., 1996), explored narratives in the form of autobiographical essays written by nuns joining a convent. That study, as well as a few other longitudinal studies, have through a prospective or a retrospective analysis linked changes in semantic and lexical content to cognitive decline or development of dementia later in life (Snowdon et al., 1996; Garrard et al., 2004; Farias et al., 2012). However, most studies rely on cross-sectional analysis to explore language features connected to cognitive decline or carry out longitudinal analysis of persons already diagnosed with some type of impairment. A review found that fluency, semantic and speech production outcome measures are most efficient when discriminating persons with MCI from controls (Filiou et al., 2020). These measures were also useful in discriminating MCI and mild AD from controls, whereas syntactic outcome measures were found to be efficient first at mild-moderate stages of the disease, which is consistent with previous studies (Kemper et al., 1993; Ahmed et al., 2013).

Despite the multiple benefits of using a more in-depth qualitative analysis, this is often discarded in a clinical setting due to time constraints. Hence, there is a need for assessment tools for analysis of continuous speech that are easy to use clinically and that can differentiate between persons with cognitive decline and normal ageing. A protocol was developed by Harris et al. (2008), also described in Kiran et al. (2005) and Fleming (2014) to measure the quality of discourse in a task designed to place high demands on executive functioning. They have also developed a protocol for assessing differences in thematic content and used it to differentiate between persons with MCI and controls, with the intent to capture changes in communicative effectiveness. It has been suggested that subtle changes in the overall communicative effectiveness may be early markers of communicative decline, and that the thematic analyses are more efficient and clinically informative than an analysis of linguistic features when evaluating communicative competence (Harris et al., 2008). This type of analysis can be viewed as a pragmatic approach, and includes an analysis of whether the produced information is relevant to the current topic. The inclusion of off-topic information indicates a disruption of discourse, and has been found to have a higher occurrence in discourse of persons with mild AD (Toledo et al., 2018). A higher occurrence was found of a similar type of disruption of coherence, called modalizations, that can be conceptualized as comments or opinions about the speaker's performance during the discourse (Toledo et al., 2018). Whereas the first study using the complex discourse task called the planning task could discriminate between the groups with regards to the thematic analysis (Harris et al., 2008), the more recent study could not (Fleming, 2014). However, both studies could discriminate persons with MCI from persons without cognitive impairment on some type of linguistic analyses, which implies that the task used is complex enough to be used in early stages of cognitive decline.

The purpose of this study is to explore how semantic impairments associated with cognitive deterioration manifest themselves in discourse, and to investigate if measures of semantic content in discourse can be used to distinguish between persons with a stable cognitive impairment (referred to as our cognitively stable group, CS-group), ongoing cognitive decline (referred to as the cognitively declined group, CD-group), and healthy controls (HC-group). To be able to test our methods used to explore semantic production in this type of task, we first needed to know if our groups differ in term of semantic ability. Hence, our first research question concerns this query. Our research questions are:

Does semantic ability (in terms of oral lexical retrieval) as measured on standardized tests differ between persons with cognitive impairment who have cognitively declined, persons with cognitive impairment who are cognitively stable, and a control group?

Do discourse features, in terms of content and disfluencies, differ between persons with cognitive impairment who have cognitively declined, or are cognitively stable in comparisons with a control group?

Can semantically related discourse features be used to improve classification accuracy when combined with SVF results in a machine learning experiment?

Our hypotheses are that:

- semantic ability as measured on standardized tests differ between persons with cognitive impairment who have cognitively declined, persons with cognitive impairment who are cognitively stable, and a control group. We expect the persons with cognitive impairment who have cognitively declined to score lower on the tests than the persons with cognitive impairment who are cognitively stable, and we expect the control group to score the highest.
- discourse features differ between persons with cognitive impairment who have cognitively declined, persons with cognitive impairment who are cognitively stable, and a control group. We expect the persons with cognitive impairment who have cognitively declined to perform worse with regards to discourse features than the persons with cognitive impairment who are cognitively stable, and we expect the control group to perform best.
- classification accuracy can be improved by adding discourse features to SVF results in a machine learning experiment.

## Method

### Participants

The participants in the study consist of 40 persons with cognitive impairment and 28 healthy controls (HC). The participants with cognitive impairment were recruited from the Gothenburg MCI study, a longitudinal study investigating dementia disorders in patients seeking medical care at a memory clinic (Wallin et al., 2016). Inclusion criteria included age 50–79 years and Swedish as their first and only language before the age of 5 years. Exclusion criteria were occurrence of other health conditions that might affect cognitive functioning, such as stroke or brain tumor, substance abuse, serious psychiatric impairment, major depression, or neurological disease. Additional reasons for exclusion were dyslexia and any uncorrected vision or hearing difficulties. The control group was recruited primarily through senior citizens' organizations, using the same exclusion criteria. They also underwent an assessment to rule out any subjective or objective cognitive impairment, and were excluded if they had a Mini Mental State Examination (MMSE; Folstein et al., 1975) score below 26. An overview of the participants is presented in Table 1, together with their scores on the MMSE, BNT (Kaplan and Weintraub, 1983), and SVF.

**Table 1:** Demographic data.

	**CD (*n* = 13) mean (SD)**	**CS (*n* = 27) mean (SD)**	**HC (*n* = 28) mean (SD)**	**Comparison**
Age	74.9 (3.6)	68.6 (6.8)	69.5 (7.3)	*p* = 0.013^*^
Education	14.9 (3.9)	14.6 (3.0)	13.3 (3.4)	*p* = 0.085
MMSE (max 30)	25.8 (3.3)	28.8 (1.9)	29.2 (1.0)	*p* ≤ 0.001^***^
BNT (max 58)	44.3 (11.1)	53.8 (3.1)	52.9 (3.9)	*p* = 0.002^**^
SVF	17.6 (4.6)	27.0 (6.2)	25.1 (6.2)	*p* ≤ 0.001^***^

* *sig. at p-level 0.05*,

** *sig. at p-level 0.01*,

*** *sig. at p-level 0.001. Note: Two persons declined testing with BNT resulting in n 12 in the CD-group and n 26 in the CS-group in this comparison*.

### Data Collection

The data collection was divided into two parts: the neuropsychological testing and cognitive/functional assessments, and the language tasks. The cognitive/functional assessment and the neuropsychological testing was administered at the memory clinic by a psychologist or a supervised research nurse. All testing was then assessed by a psychologist (ME). The examination was performed in two sessions of 1.5–2 h. Neuropsychological testing and cognitive assessment was carried out before the collection of language data and again after the language data collection had been completed.

Participants took part in collection of language data at two dates ~18 months apart, and this study is based on data from the second data collection. The administration of the language tasks took place in a quiet lab environment at University of Gothenburg. The participants completed a discourse task, the SVF as well as some additional tasks not analyzed in the present study.

The first collection of language data included 91 participants, of which 55 persons were diagnosed with some type of cognitive impairment (MCI or SCI) and 36 HC matched for age and education. At the second collection of language data 21 persons failed to return for various reasons. Additionally, one person was excluded due to poor sound quality in the recordings of the language tasks and HC person was excluded due to an MMSE score below 26 at the renewed cognitive assessment.

### Neuropsychological Testing and Assessment of Cognitive Status

All participants underwent neuropsychological testing. The participants with cognitive impairment also underwent a cognitive/functional assessment to determine the level of impairment. The tests were selected by clinical neuropsychologists at the memory clinic based on the tests' documented ability to predict subsequent dementia (Eckerström et al., 2013), and with the aim to cover a broad cognitive spectrum. The level of cognitive impairment was assessed with the Global Deterioration Scale (GDS-scale; Auer and Reisberg, 1997) based on four instruments: MMSE (Folstein et al., 1975), Clinical dementia rating (CDR), Stepwise comparative status analysis (Wallin et al., 1996), and I-FLEX (short version of Executive interview EXIT; Royall et al., 1992).

The neuropsychological test battery included tests of learning and memory, language, attention, and executive function. For learning and memory, Rey Auditory Verbal Learning Test (Geffen et al., 1994), Rey Complex Figure (Meyers and Meyers, 1995), recalled after 3 and 20 min, and Weschler Logical Memory subtest (Wechsler, 2003) were used. For language, Boston Naming Test (Kaplan and Weintraub, 1983), verbal fluency for letters F-A-S (Lezak et al., 2012), similarities subtest from the Wechsler Adult Intelligence Scale (WAIS; Wechsler, 2003) and the Token Test, part 5 (De Renzi and Vignolo, 1962) were used. For attention WAIS Digit Span test, WAIS Digit-Symbol test (Wechsler, 2003), the Trail-Making Test forms A and B (Reitan and Wolfson, 1985), for visuo-spatial ability WAIS Block Design test (Wechsler, 2003), Rey Complex Figure copy, and Silhouettes subtest from the Visual Object and Space Perception Battery (Binetti et al., 1996) were used. Finally, for executive function WAIS Letter-Number sequencing subtest, Parallel Serial Mental Operations (Lezak et al., 2012), and the Stroop test (Regard, 1981) were used. All testing was then assessed by a psychologist (ME).

After the second cognitive assessment, the participants with cognitive impairment were divided into those who had deteriorated since the first assessment, the cognitive decline group (CD, n 13) and those who not had deteriorated, the cognitively stable group (CS, n 27). This categorisation was based both on the cognitive assessment and the neuropsychological testing. Six patients converted from mild cognitive impairment to dementia (i.e., scored GDS 3 at baseline and GDS 4/4+ at follow-up). Another seven patients declined cognitively during the study time, based on neuropsychological testing, but did not fulfill criteria for dementia. When analysing neuropsychological test scores, the cut-off for “cognitively impaired” was set at 1.5 standard deviations below the normal mean. Patients had to score below cut-off on at least one out of the nine test variables. The normal mean scores were calculated based on scores from cognitively healthy volunteers included in the Gothenburg MCI study (*n* = 117), and were controlled for significant differences based on age and years of education. Cognitive decline was based on each patient's number of test variables in the normal vs non-normal range (i.e., using the 1.5 standard deviations cut-off). Cognitive decline was defined as a decline (i.e., changed score from normal to below-normal range) from baseline to follow-up in two or more neuropsychological test variables.

### Tests of Semantic Ability

The performance on the SVF with the category “animals” (part of the language data collection) and the BNT (Kaplan and Weintraub, 1983) (part of the neuropsychological tests) were used as baseline measures of semantic ability. Administration and scoring was done in accordance with (Tallberg et al., 2008) for SVF and (Tallberg, 2005) for BNT. Due to inconsistent scoring on two items in BNT, these two items were excluded resulting in a maximum of 58 points instead of 60.

### Discourse Task

The spontaneous language material analyzed in the present study consists of a spoken discourse task, which was modeled on the “Trip to New York” task developed and validated by Kiran et al. (2005), and described in Harris et al. (2008). For the purposes of this project, the task was changed to “Trip to Stockholm.” The participants were asked to describe how they would prepare for and execute a trip to Stockholm. The instructions were as follows:

Now you are going to do a task where you are asked to think and plan aloud. Imagine that you are going on a vacation a week from now. You are traveling to Stockholm for a 2-week stay. Think about all you will have to do to get ready to go, such as how you will get there, what you will bring, and what you will do. I want you to tell me all of your plans until I ask you to stop after about 5 min.

A few follow-up questions were posed if they had not mentioned this information in their narratives, such as: Who will take care of your mail? What will you bring on your trip? The planning task was designed to elicit connected language, that required the participant to supply conceptual and semantic content related to the cognitive-linguistic schema for travel (Harris et al., 2008). It is further suggested to be complex enough to reveal subtle changes in persons with brain damage, due to its demands on executive functions such as initiation, planning, temporal organization and flexibility, and also semantic, episodic and working memory processes.

### Data Preparation

The recordings were transcribed orthographically by two certified speech-language pathologists who transcribed approximately half of the recordings each. The transcribers were instructed to segment the discourse into sentences. A clause was defined as having to contain one finite verb, and a sentence defined as consisting of one or several clauses. Besides considering the clauses, the segmentation was based on the speakers' prosodic markers that could indicate sentence breaks. For example, falling intonation could indicate the end of an utterance and thus marked a sentence break. The transcribers trained together before transcribing the participants' recordings to ensure that they interpreted the transcription key correctly. Additionally, each recording was checked twice by one of the transcribers (the first author).

To make the linguistic analysis more efficient, methods from the field of language technology were used. The transcriptions were annotated with part-of-speech (POS) tags and each word was lemmatized using Sparv (Borin et al., 2016). Alignment of the audio recordings and transcriptions was made using Webmaus (Kisler et al., 2017), with post-corrections done manually.

### Linguistic Analyses of Discourse Task

The discourse task was analysed with regard to themes and disfluencies, as described in the following sections. Furthermore, some basic narrative characteristics are presented in Table 2. Total phonation duration is the total time spent speaking excluding silent pauses.

**Table 2:** Comparison of basic narrative characteristics for the discourse task per group.

	**CD mean (SD)**	**CS mean (SD)**	**HC mean (SD)**	**Comparison**
N words	334 (122.9)	439 (201.4)	412 (161.7)	*p* = 0.187
N full sentences	22.4 (7.3)	28.7 (12.5)	29.8 (12.0)	*p* = 0.1
N words per sentences	14.0 (4.3)	14.7 (4.1)	14.1 (5.0)	*p* = 0.653
Total phonation duration	107.1 (36.2)	132.6 (61.8)	117.7 (46.7)	*p* = 0.512

Note: *sig. at p-level 0.05, **sig. at p-level 0.01, ***sig. at p-level 0.001.

#### Semantic Content

To capture semantic aspects of discourse, we focused on thematic content and modalizing language. Modalizations are sometimes referred to as metadiscourse and can be described as remarks on the content of the story e.g., “yeah I can't think of anything else at the moment that I want to do,”^1^ and/or concerns about its production (Farias et al., 2012; Toledo et al., 2018) e.g., “…but I always forget what it is called”^1^ or “no by the way that's not correct.”^1^ The thematic coding was based on a previously validated protocol (Harris et al., 2008) used in several studies on the same population (Kiran et al., 2005; Harris et al., 2008; Fleming, 2014). The coding protocol consists of 13 defined core elements i.e., different subtopics/themes: temporal, transportation/ticket, work school/family, money/cost, clothing/packing, lodging, medication/health, securing/housing, activities, food, people, identification, and local cost/money. These were rated 0 if not mentioned, 1 if mentioned briefly, and 2 if elaborated upon. Verbosity or irrelevant comments resulted in a deduction: −1 if minimally present and −2 if significantly present. Minimally present was defined as one irrelevant comment and significantly present was defined as several irrelevant comments or a longer segment of irrelevant information or verbosity. If a theme was mentioned only after the participant was asked a question about that theme, no point was given. Besides scoring the texts according to Fleming (2014), additional analyses of the themes included analysing the number and proportion of words coded as themes, words coded as modalizations and words coded as unrelated speech (i.e., irrelevant comments).

#### Disfluencies

Disfluencies are related to the process of planning and producing language. Four types of disfluencies were annotated and analyzed: silent pauses, fillers, false starts, and self-interrupted sentences. Silent pauses were defined as an interval >120 ms within the discourse that is not filled with speech or other sounds produced by the speakers, such as coughing or laughing. The 120 ms cut-off was chosen based on the detection threshold for acoustic silences in speech (Heldner, 2011). Fillers were defined as sounds that indicate e.g., hesitation or planning but that do not have lexical content. Examples of fillers include “uh” and “um.” A false start means that the person has started articulating a word, but did not complete it; e.g., the persons says “I pa- pack shoes.”^1^ Self-interrupted sentences are sentences where the person started producing a sentence but did not complete it; e.g., the person says “and then you could take some—maybe there is some sightseeing- e thing with bus or something like that.”^1^ If several disfluencies occurred in a row, they were handled as separate instances. The number of disfluencies present in the speech of the participants were measured, as well as the duration of pauses and fillers.

### Classification Experiment

To evaluate the usefulness of the extracted features, we tested whether adding them to the SVF score in a machine learning model would improve classification of participants as cognitively stable or cognitively deteriorating. The classification experiment was implemented in Python and Scikit-learn (Pedregosa et al., 2011). For the classification experiment, three common machine learning models used for supervised classification were used: Support Vector Machines (SVM), Gaussian NaiveBayes (NB), and Logistic Regression (LR). Feature selection was performed with SelectKBest, which keeps the n highest scoring features based on an evaluation with an ANOVA. Leave-one-out cross-validation was used for all models. Features were standardized according to the training set in each fold (except for NaiveBayes, since it is invariant to feature scaling), and default hyper-parameters were used. For evaluation, we use area under the receiver operating characteristics curve (AUC). The AUC is calculated by plotting sensitivity (true positive rate) against false positive rate (1 – specificity), as the decision threshold of the classifier is varied. The area under the resulting curve is the AUC, and the better the model is at classifying the groups, the higher is the resulting AUC.

### Statistical Methods

Non-parametric tests were chosen as the groups were rather small, and many of the variables were skewed. Kruskal-Wallis were used to compare differences between the groups and Mann-Whitney *U*-for independent samples were used for *post-hoc* analyses. A more stringent significance level was adopted due to multiple comparisons. After the Bonferroni corrections the new alpha level was *p* = 0.01 for the comparisons of the lexical features (the basic narrative characteristics presented in Table 2), *p* = 0.006 for comparisons of thematic content and modalizations and *p* = 0.006 for comparisons of disfluencies. We chose to report both at a significance level of *p* = 0.05 and at the Bonferroni-corrected level. Since there was a significant difference between the groups in age, where the CD group was significantly older than the other two groups, age was added as a covariate in a univariate linear model (ANCOVA) to explore the effect of age. This was only done when there was a relationship between age and the tested variable. Since ANCOVA is a parametric test the dependent variables were logtransformed to meet the assumption of normality. IBM SPSS Statistics version 25 and 26, and R version 3.6.1 (R Core Team, 2019) were used as computational tools.

### Ethical Considerations

The present study is covered by the ethical approval (reference number: 206–16, 2016; T021-18) issued by the regional ethical review board in Gothenburg for a larger project. The participants were informed that they could withdraw their participation at any time. All data was coded and made anonymous.

## Results

### Tests of Semantic Ability

There were significant differences between the groups on both BNT and SVF, see Table 1. *Post-hoc* analyses revealed that the CD group had a significantly lower result than the other two groups on both tests. An ANCOVA was performed to explore the effect of age on the results. Both comparisons were still significant after adjusting for age: BNT *F*_(2, 62)_ = 7.48, *p* = 0.002, SVF *F*_(2, 64)_ = 8.21, *p* = 0.001.

### Analysis of Discourse Task

Basic narrative characteristics of the discourse task are provided for groups in Table 2. The groups did not differ significantly on the number of words and sentences produced or on total phonation duration.

### Semantic Content

The difference between the groups in the thematic content score was borderline significant (see Table 3 for an overview of all comparisons related to the thematic analysis) A *post-hoc* analysis revealed a difference between the CD group and HC group (U = 99.5, *p* = 0.019), but not between the CD group and the CS group (U = 116, *p* = 0.08). Since the thematic content score correlated with age, an ANCOVA was performed with the CD group and the controls added as independent variables, to evaluate the effect of age. Age had a significant effect whereas no effect was seen on the group variable [*F*_(1, 38)_ = 2.20; *p* = 0.15], suggesting that age and not group explained the difference in thematic content score in this comparison. The number of words in themes were significantly different between the groups (at level *p* < 0.05), but not in the comparison of the proportion of words in themes, indicating that when adjusting for the total number of words in each narrative the proportion of how much they talked about the trip planning was similar. None of the features measuring modalizing speech differed between the groups. Unrelated speech was used rarely, less than once per participant (CD M = 0.46, CD M = 0.07, and HC M = 0.18), but most often for the CD group. A *post-hoc* analysis revealed that the CD group produced a higher proportion of unrelated speech than the CS group (U = 112, *p* = 0.003), and the HC group (U = 129, *p* = 0.032). Only the difference in proportion of unrelated speech survived the Bonferroni corrected p-level.

**Table 3:** Comparisons of thematic content and modalizations.

	**CD mean (SD)**	**CS mean (SD)**	**HC mean (SD)**	**Comparison**
Thematic content score	8.2 (2.5)	9.8 (2.8)	10.6 (2.9)	*p* = 0.052
Words in themes (*n*)	192.8 (103.6)	179.3 (34.5)	290.1 (134.8)	*p* = 0.046^*^
Words in themes (%)	76.4 (23.0)	87.6 (12.8)	89.4 (10.4)	*p* = 0.156
Modalizations (*n*)	0.8 (0.6)	1.0 (0.9)	1.1 (0.9)	*p* = 0.642
Modalizations (*n* of words)	6.6 (8.2)	11.0 (13.2)	11.4 (14.1)	*p* = 0.343
Modalizations (%)	3.1 (4.2)	3.9 (5.6)	4.1 (4.1)	*p* = 0.501
Unrelated speech (*n*)	0.46 (0.66)	0.07 (0.39)	0.18 (0.61)	*p* = 0.013^*^
Unrelated speech (*n* of words)	30.3 (46.3)	1.6 (8.5)	9.9 (32.9)	*p* = 0.009^*^
Unrelated speech (%)	13.0 (23.3)	0.4 (2.2)	2.0 (6.7)	***p*** **=** 0.006^*^

*Note*:

* sig. at p-level 0.05, **sig. at p-level 0.01, ***sig. at p-level 0.001. Bold type numbers are significant at the Bonferroni corrected alpha level p ≤ 0.006.

### Disfluencies

Disfluencies in the narratives of the participants were analyzed, and results (see Table 4) showed that the groups differ significantly with regard to the number of pauses used (normalized by number of words), the maximum length of pauses and on the total number of disfluencies, i.e., silent pauses, fillers, false starts and self-interruptions, used (normalized by number of words). However, none of the significant results survived a Bonferroni correction.

**Table 4:** Analysis of disfluency features.

	**CD mean (SD)**	**CS mean (SD)**	**HC mean (SD)**	**Comparison**
Silent pauses per 100 words	18.8 (7.7)	14.8 (3.0)	12.5 (3.5)	*p* = 0.01^**^
Fillers per 100 words	2.6 (1.6)	3.9 (2.3)	3.1 (2.0)	*p* = 0.203
False starts per 100 words	0.54 (1.6)	0.70 (0.70)	1.0 (1.1)	*p* = 0.220
Self-interrupted sentences per total sentences	0.18 (0.12)	0.17 (0.10)	0.14 (0.10)	*p* = 0.294
Disfluencies per 100 words	23.1 (8.3)	20.5 (3.9)	17.7 (5.1)	*p* = 0.017^*^
Mean pause length (>120 ms)	0.78 (0.21)	0.74 (0.18)	0.71 (0.30)	*p* = 0.138
Maximum pause length (>120 ms)	3.5 (1.7)	3.0 (1.5)	2.4 (1.3)	*p* = 0.007^**^
Mean filler length	0.45 (0.16)	0.50 (0.15)	0.43 (0.1)	*p* = 0.151

*Note*:

* *sig. at p-level 0.05*,

** sig. at p-level 0.01, ***sig. at p-level 0.001. Bold type numbers are significant at the Bonferroni corrected alpha level p ≤ 0.006.

*Post-hoc* analyses show that persons with cognitive decline and persons who were cognitively impaired but stable did not differ from each other with regard to any of the significant disfluency measures. However, both groups differed significantly from the healthy controls.

### Classification Experiment

We evaluated the predictive accuracy of different collections of features by using them in a machine learning model. Since we in this experiment were interested in separating the CS-group from the CD-group) only features from these two groups were applied in the model. The results are presented in Table 5. As a baseline, we trained the model using only the results from the SVF, as impairments on the SVF have been found to be predictive of dementia (Taler and Phillips, 2008; Belleville et al., 2017). Using only this feature, we achieved a best result of AUC = 0.86 with SVM. We then added the lexical content features, the semantic features and the fluency features separately to the SVF results, and found that this led to improved results, except when training on the SVF and the lexical features and using the SVM classifier, which gave the same AUC as only training on the SVF. Finally, we trained a model using all features combined. The features found most useful were a combination of SVF results and the disfluency features, and training on this data gave the best results for all three classifiers, with the SVM achieving the highest AUC result of 0.93.

**Table 5:** Area under the curve results for the 3 machine learning algorithms with different combinations of features.

	**SVM**	**LR**	**NB**
SVF	0.86	0.82	0.78
SVF + lexical features	0.86	0.89	0.87
SVF + semantic features	0.87	0.89	0.87
SVF + disfluency features	**0.93**	0.91	0.90
SVF + all features	0.87	0.89	0.87

*The boldfaced number indicates the best result. SVF, semantic verbal fluency; SVM, Support Vector Machines; LR, Logistic Regression; NB, Gaussian NaiveBayes*.

## Discussion

The present study aimed to investigate semantic aspects of discourse produced by persons who declined cognitively, were cognitively impaired but stable, and healthy controls. To further capture their semantic production, quantitative measures of semantic ability were assessed with tests of oral lexical retrieval. These methods were used in order to explore which measures that best could discriminate between the groups. In sum, both types of assessment methods captured differences between the groups, but the tests of oral lexical retrieval most successfully differentiated between the cognitively stable and the cognitively declined group. This supports previous research which has shown that especially the SVF is a robust predictor of cognitive decline (Taler and Phillips, 2008; Belleville et al., 2017).

To explore semantic aspects of discourse we used a thematic analysis of content (including modalizations and unrelated speech) and an analysis of disfluencies. The elicitation task and the analysis of thematic content were based on the same protocol as Harris et al. (2008) and Fleming (2014). When comparing our CD group with the participants with MCI in Harris et al. (2008) and Fleming (2014), our results are similar to those of Harris et al. (2008) who found that persons with MCI provided less thematic information than the older healthy controls included in the study, and had more irrelevant comments and verbosity. The presence of content not related to the subject or modalizing speech have been found in previous studies investigating discourse in persons with MCI and mild AD (Duong et al., 2003; Drummond et al., 2015; Pistono et al., 2018; Toledo et al., 2018), and is proposed to be related to problems in the semantic-pragmatic component of the language (Drummond et al., 2015). It is further suggested to be a pragmatic ability in AD patients to be able to comment on their communicative production and that it should be viewed as a communicative strength (Duong et al., 2003, Pistono et al., 2018). Why differences in modalizations are not seen in the present study is not clear, but could perhaps be explained by that the use of a more free discourse task did not evoke as many modalizations as picture based task would do. Another possible explanation could be that the present participants' difficulties were too subtle to reveal a difference in modalizing language as seen in previous studies. The proportion of unrelated speech was the only measure that could differentiate between the group who had cognitively declined and the cognitively stable, however, there was a very low occurrence of unrelated speech. The analysis of disfluencies revealed the largest differences between the groups, and we found that the healthy controls tended to use fewer silent pauses, shorter maximum pause lengths and fewer disfluencies in total compared to the cognitively impaired groups. This result is in line with previous research showing that disfluencies are more common in discourse produced by persons with early MCI (Mueller et al., 2018) and in persons with a clinical diagnosis of MCI (Fleming, 2014; Szatloczki et al., 2015; Meilán et al., 2020).

The last research question concerned if the discourse features could improve classification accuracy when combined with SVF results in a machine learning experiment. Our focus here was distinguishing between persons who are cognitively impaired and showing progressive decline, as opposed to persons with stable cognitive impairment. The best classification results were attained by combining the SVF results with the disfluency features, which had a higher AUC (0.93 using Support Vector Machines) than using only SVF. Based on this, we draw the conclusion that the analysis of disfluencies in connected speech provide complementary information to the results on the SVF, possibly because disfluency features do not solely depend on semantic aspects of language but also executive functions which are known to be impaired in MCI (Gauthier et al., 2006).

The task used in the present study was designed to be more cognitively-linguistic challenging, and was added for the second data collections, since previous experiences from using the cookie theft picture as elicitation, suggested that a more challenging task was needed (Lundholm et al., 2018). The planning task was developed by Kiran et al. (2005) with the intent to stimulate connected language instead of more list-like labeling which sometimes can be the case in picture descriptions, and be sensitive to differences in discourse production. Previous studies suggest that task complexity is important when assessing mild impairments as in the case of early AD (Forbes et al., 2002). However, to our knowledge no study has compared the planning task to another type of task, so we can only rely on theoretical assumptions and previous studies concerning the tasks suitability. The thematic analysis was based on the protocol developed by Harris et al. (2008) and consisted of a scoring system where points were given if a certain core element was mentioned. The benefits of this scoring protocol are that it is relatively easy and quick to analyse. A critique might be that it is a bit crude. For that reason, we also chose to analyse how much the participants talked about things related to the themes (or not related to the themes), and how fluently they talked. This seems to complement the scoring, but would be quite cumbersome to implement in the clinic. For at least some of these findings, such as the importance of temporal analysis (disfluencies), they might be implemented in other tasks such as measuring latencies in BNT, or temporally resolved measures on the SVF (Linz et al., 2019). Another adjustment in the present study was the addition of follow-up questions which were posed if certain elements of the trip were not mentioned. Since the information following these questions were prompted and not mentioned spontaneously, we decided to disregard this information in the scoring. This departure from the original protocol means that our results are not completely comparable to previous studies, and we suggest excluding follow-up questions in future studies if the main outcome measure is the score of thematic content.

A drawback of this type of discourse task when used in the clinic is that it requires manual transcription. In some languages it might be possible to use automatic speech recognition, but for Swedish we did not judge the currently available speech recognition alternatives good enough for this purpose. To avoid manual transcription, the test persons can be asked to describe their trip in writing instead, which may be tested in future studies. In the present study, we used methods from language technology and computational linguistics in order to automate some of the analysis and to test if the discourse measures could improve the classification. Studies mixing manual and automated methods seems to be more and more common in this field and can hopefully complement each other (Boschi et al., 2017). Although most studies use manual transcription and segmentation, annotation with part-of-speech taggers and linguistic analyses with for example parsers are often used to make the analysis more efficient and consistent.

A question raised when using this discourse task may be that, if the tests of lexical retrieval were better at discriminating between the groups, then why not use them instead of a discourse task. However, a task that assesses functional language has a higher ecological validity than a psychometric language test (Bastiaanse and Prins, 2004), and can be more challenging, thus more suitable for subtle impairments. Related to that, Drummond et al. (2015) argued that it is often in narrative discourse elderly persons with cognitive deterioration first experience language problems that they perceive as related to impaired memory, such as repetitions or information gaps in their narratives. Furthermore, since tests such as naming and SVF do not address such discourse, deficits occurring in narrative discourse may go undetected. On the contrary, it is also possible that persons with mild difficulties are able to compensate for their problems with lexical retrieval, seen in naming or SVF tests, in a discourse task. However, even if mild word retrieval difficulties do not always lead to anomia, it might lead to an increase in pauses and other types of disfluencies, which was also the case in our data.

One limitation in the present study was the rather small sample, especially in the group with persons who declined cognitively. At the start of the longitudinal project that this study is a part of, participants with either SCI or MCI were included, but due to dropouts the groups ended up rather small at the second point of data collection. The sample size may explain why so few of the comparisons survived Bonferroni adjustments, even though there was a difference in rank seen at alpha level 0.05.

We chose to categorize the participants with a cognitive impairment, according to if they had declined or not from the time when they were included in the project in order to explore which aspects are related to cognitive deterioration. A consequence of this categorisation was that the group with persons who had cognitively declined had a higher age than the persons who were stable and the controls. Since the risk of cognitive impairment increases with age (Unverzagt et al., 2001), it is not surprising that our groups have these demographic characteristics. However, we decided to adjust the comparisons for this factor in those comparisons were there was a relationship between the dependent variable and age. In the case of BNT and SVF, the difference in results were still significant, but not for the difference in thematic content score seen between the CD group and controls.

In sum, the tasks complement each other where the standardized tests provide easy administration and analysis while the planning task offers a more ecologically valid evaluation of spoken language. The tests will indicate which words the persons struggle to find, whereas a discourse task may also reveal what strategies the persons use when experiencing word finding difficulties, and how they are able to compensate. With a larger number of participants, differences between the groups in the discourse task may become more distinct, but differences in communicative efficacy (thematic content score) and fluency seems the most promising variables for future work.

Although the project that this study is a part of is longitudinal, data on the planning task is only available from the second data collection, since it was included later in order to add tasks with a higher complexity. Longitudinal data on this task is needed in order to find out if discourse features such as the ones used in the present study really are useful predictors of cognitive decline.

## Data Availability Statement

The datasets presented in this article are not readily available because they contain sensitive and personally-identifying information. Requests to access the datasets should be directed to Dimitrios Kokkinakis, dimitrios.kokkinakis@svenska.gu.se.

## Ethics Statement

The studies involving human participants were reviewed and approved by the regional ethical review board in Gothenburg (reference number: 206–16, 2016; T021-18). The patients/participants provided their written informed consent to participate in this study.

## Author Contributions

KL and DK designed the overall study protocol and collected the data. MA and KL were responsible for developing the research questions for this study, conducting all linguistic analyses, and wrote the first draft of the paper. ME provided the neuropsychological scores and contributed to the clinical interpretation. KL designed and implemented the classification experiments. All authors contributed to manuscript revision, read, and approved the submitted version.

## Conflict of Interest

The authors declare that the research was conducted in the absence of any commercial or financial relationships that could be construed as a potential conflict of interest.

## Footnotes

^1^Examples taken from the data (translated from Swedish).

## References

[B1] Ahmed S. Haigh A. F. de Jager C. Garrard P. (2013). Connected speech as a marker of disease progression in autopsy-proven alzheimer's disease. Brain 136(Pt 12), 3727–3737. 10.1093/brain/awt269 24142144 PMC3859216

[B2] Ahmed S. Mitchell J. Arnold R. Nestor P. J. Hodges J. R. (2008). Predicting rapid clinical progression in amnestic mild cognitive impairment. Dement. Geriatr. Cogn. Disord. 25, 170–177. 10.1159/000113014 18212499

[B3] Auer S. Reisberg B. (1997). The GDS/FAST staging system. Int. Psychogeriatr. 9(Suppl. 1), 167–171. 10.1017/S1041610297004869 9447440

[B4] Auriacombe S. Lechevallier N. Amieva H. Harston S. Raoux N. Dartigues J. (2006). A longitudinal study of quantitative and qualitative features of category verbal fluency in incident alzheimer's disease subjects: results from the PAQUID study. Dement. Geriatr. Cogn. Disord. 21, 260–266. 10.1159/000091407 16465054

[B5] Bastiaanse R. Prins R. (2004). Review: analysing the spontaneous speech of aphasic speakers. Aphasiology 18, 1075–1091. 10.1080/02687030444000534

[B6] Belleville S. Fouquet C. Hudon C. Zomahoun H. T. V. Croteau J. (2017). Neuropsychological measures that predict progression from mild cognitive impairment to alzheimer's type dementia in older adults: a systematic review and meta-analysis. Neuropsychol. Rev. 27, 328–353. 10.1007/s11065-017-9361-5 29019061 PMC5754432

[B7] Binetti G. Cappa S. F. Magni E. Padovani A. Bianchetti A. Trabucchi M. (1996). Disorders of visual and spatial perception in the early stage of alzheimer's disease. Ann. N. Y. Acad. Sci. 777, 221–225. 10.1111/j.1749-6632.1996.tb34422.x 8624088

[B8] Borin L. Forsberg M. Hammarstedt M. Rosén D. Schäfer R. Schumacher A. (2016). “Sparv: språkbanken's corpus annotation pipeline infrastructure,” in The Sixth Swedish Language Technology Conference (SLTC) (Umeå: UmeåUniversity), 17–18.

[B9] Boschi V. Catricalà E. Consonni M. Chesi C. Moro A. Cappa S. F. (2017). Connected speech in neurodegenerative language disorders: a review. Front. Psychol. 8:269. 10.3389/fpsyg.2017.00269 28321196 PMC5337522

[B10] Carswell L. M. (1999). Prediction of memory and language performance in normal elderly canadians: implications for the assessment of premorbid cognition in early alzheimer's disease. Dissert. Abstract. Int. B Sci. Eng. 60:2935 10.1093/arclin/14.8.670a

[B11] Clark L. J. Gatz M. Zheng L. Chen Y. McCleary C. Mack W. J. (2009). Longitudinal verbal fluency in normal aging, preclinical, and prevalent alzheimer's disease. Am. J. Alzheimer's Dis. Other Dement. 24, 461–468. 10.1177/1533317509345154 19759254 PMC2824246

[B12] Davis B. H. Maclagan M. (2009). Examining pauses in alzheimer's discourse. Am. J. Alzheimer's Dis. Other Dement. 24, 141–154. 10.1177/1533317508328138 19150969 PMC10846024

[B13] De Renzi E. Vignolo L. A. (1962). Token test: a sensitive test to detect receptive disturbances in aphasics. Brain 85, 665–678. 10.1093/brain/85.4.665 14026018

[B14] Demetriou E. Holtzer R. (2017). Mild cognitive impairments moderate the effect of time on verbal fluency performance. J. Int. Neuropsychol. Soc. 23, 44–55. 10.1017/S1355617716000825 27748216 PMC5325672

[B15] Drummond C. Coutinho G. Paz Fonseca R. Assunção N. Teldeschi A. de Oliveira-Souza R.. (2015). Deficits in narrative discourse elicited by visual stimuli are already present in patients with mild cognitive impairment. Front. Aging Neurosci. 7:96. 10.3389/fnagi.2015.00096 26074814 PMC4446997

[B16] Duong A. Tardif A. Ska B. (2003). Discourse about discourse: what is it and how does it progress in alzheimer's disease? Brain Cogn. 53, 177–180. 10.1016/S0278-2626(03)00104-0 14607142

[B17] Eckerström C. Olsson E. Bjerke M. Malmgren H. Edman Å. Wallin A.. (2013). A Combination of neuropsychological, neuroimaging, and cerebrospinal fluid markers predicts conversion from mild cognitive impairment to dementia. J. Alzheimer's Dis. 36, 421–431. 10.3233/JAD-122440 23635408

[B18] Engelhardt P. E. McMullon M. E. G. Corley M. (2019). Individual differences in the production of disfluency: a latent variable analysis of memory ability and verbal intelligence. Q. J. Exp. Psychol. 72, 1084–1101. 10.1177/1747021818778752 29756526

[B19] Farias S. T. Chand V. Bonnici L. Baynes K. Harvey D. Mungas D.. (2012). Idea density measured in late life predicts subsequent cognitive trajectories: implications for the measurement of cognitive reserve. J. Gerontol. B Psychol. Sci. Soc. Sci. 67, 677–686. 10.1093/geronb/gbr162 22357642 PMC3478727

[B20] Filiou R. Bier N. Slegers A. Houzé B. Belchior P. Brambati S. M. (2020). Connected speech assessment in the early detection of alzheimer's disease and mild cognitive impairment: a scoping review. Aphasiology 34, 723–755. 10.1080/02687038.2019.1608502

[B21] Fleming V. B. (2014). Early detection of cognitive-linguistic change associated with mild cognitive impairment. Commun. Disord. Q. 35, 146–157. 10.1177/1525740113520322

[B22] Folstein M. F. Folstein S. E. McHugh P. R. (1975). ‘Mini-mental state'. A practical method for grading the cognitive state of patients for the clinician. J. Psychiatr. Res. 12, 189–198. 10.1016/0022-3956(75)90026-6 1202204

[B23] Forbes K. E. Venneri A. Shanks M. F. (2002). Distinct patterns of spontaneous speech deterioration: an early predictor of alzheimer's disease. Brain Cogn. 48, 356–361. 10.1006/brcg.2001.1377 12030467

[B24] Gallagher D. Ni Mhaolain A. Coen R. Walsh C. Kilroy D. Belinski K.. (2010). Detecting prodromal alzheimer's disease in mild cognitive impairment: utility of the CAMCOG and other neuropsychological predictors. Int. J. Geriatr. Psychiatry 25, 1280–1287. 10.1002/gps.2480 21086538

[B25] Garrard P. Maloney L. M. Hodges J. R. Patterson K. (2004). The effects of very early alzheimer's disease on the characteristics of writing by a renowned author. Brain 128, 250–260. 10.1093/brain/awh341 15574466

[B26] Gauthier S. Reisberg B. Zaudig M. Petersen R. C. Ritchie K. Broich K.. (2006). Mild cognitive impairment. Lancet 367, 1262–1270. 10.1016/S0140-6736(06)68542-5 16631882

[B27] Gayraud F. Lee H. Barkat-Defradas M. (2011). Syntactic and lexical context of pauses and hesitations in the discourse of alzheimer patients and healthy elderly subjects. Clin. Linguist. Phon. 25, 198–209. 10.3109/02699206.2010.521612 21080826

[B28] Geffen G. M. Butterworth P. Geffen L. B. (1994). Test-retest reliability of a new form of the auditory verbal learning test (AVLT). Arch. Clin. Neuropsychol. 9, 303–316. 10.1093/arclin/9.4.303 14589623

[B29] Harris J. L. Kiran S. Marquardt T. Fleming V. (2008). Communication wellness check-up©: age-related changes in communicative abilities. Aphasiology 22, 813–825. 10.1080/02687030701818034

[B30] Heldner M. (2011). Detection thresholds for gaps, overlaps, and no-gap-no-overlaps. J. Acoust. Soc. Am. 130, 508–513. 10.1121/1.3598457 21786916

[B31] Herbert R. Hickin J. Howard D. Osborne F. Best W. (2008). Do picture-naming tests provide a valid assessment of lexical retrieval in conversation in aphasia? Aphasiology 22, 184–203. 10.1080/02687030701262613

[B32] Johnson S. C. Koscik R. L. Jonaitis E. M. Clark L. R. Mueller K. D. Berman S. E.. (2018). The wisconsin registry for alzheimer's prevention: a review of findings and current directions. Alzheimer's Dement. 10, 130–142. 10.1016/j.dadm.2017.11.007 29322089 PMC5755749

[B33] Kaplan H. Weintraub S. (1983). The Boston Naming Test, 2nd Edn. Philadelphia, PA: Lea and Febiger.

[B34] Kemper S. LaBarge E. Ferraro F. R. Cheung H. Cheung H. Storandt M. (1993). On the preservation of syntax in alzheimer's disease: evidence from written sentences. Arch. Neurol. 50, 81–86. 10.1001/archneur.1993.00540010075021 8418805

[B35] Kim B. S. Kim Y. B. Kim H. (2019). Discourse measures to differentiate between mild cognitive impairment and healthy aging. Front. Aging Neurosci. 11:221. 10.3389/fnagi.2019.00221 31507406 PMC6714864

[B36] Kiran S. Harris J. L. Marquardt T. P. (2005). Communication Wellness Check-Ups: Age-Related Changes in Communication. San Diego, CA: ASHA National Convention.

[B37] Kisler T. Reichel U. Schiel F. (2017). Multilingual processing of speech via web services. Comput. Speech Lang. 45, 326–347. 10.1016/j.csl.2017.01.005

[B38] Lezak M. D. Howieson D. Loring D. (2012). Neuropsychological Assessment, 5th Edn. Oxford: Oxford University Press.

[B39] Linz N. Lundholm Fors K. Lindsay H. Eckerström M. Alexandersson J. Kokkinakis D. (2019). “Temporal analysis of the semantic verbal fluency task in persons with subjective and mild cognitive impairment,” in Proceedings of the Sixth Workshop on Computational Linguistics and Clinical Psychology, eds K. Niederhoffer, K. Hollingshead, P. Resnik, R. Resnik, and K. Loveys (Stroudsburg, PA, USA: Association for Computational Linguistics), 103–113.

[B40] Loewenstein D. A. Greig M. T. Schinka J. A. Barker W. Shen Q. Potter E.. (2012). An investigation of PreMCI: subtypes and longitudinal outcomes. Alzheimer's Dementia 8, 172–179. 10.1016/j.jalz.2011.03.002 22546351 PMC3341845

[B41] Lundholm F. K. Fraser K. Kokkinakis D. (2018). Automated Syntactic Analysis of Language Abilities in Persons with Mild and Subjective Cognitive Impairment. Stud Health Technol Inform. 247, 705–709 10.3233/978-1-61499-852-5-705 29678052

[B42] Mazzon G. Ajčević M. Cattaruzza T. Menichelli A. Guerriero M. Capitanio S.. (2019). Connected speech deficit as an early hallmark of CSF-defined alzheimer's disease and correlation with cerebral hypoperfusion pattern. Curr. Alzheimer Res. 16, 483–494. 10.2174/1567205016666190506141733 31057108

[B43] McCullough K. C. Bayles K. A. Bouldin E. D. (2019). Language performance of individuals at risk for mild cognitive impairment. J. Speech Lang. Hear. Res. 62, 706–722. 10.1044/2018_JSLHR-L-18-0232 30950734

[B44] Meilán J. J. G. Martínez-Sánchez F. Martínez-Nicolás I. Llorente T. E. Carro J. (2020). Changes in the rhythm of speech difference between people with nondegenerative mild cognitive impairment and with preclinical dementia. Behav. Neurol. 2020:4683573. 10.1155/2020/4683573 32351632 PMC7178534

[B45] Mendonça M. D. Alves L. Bugalho P. (2016). From subjective cognitive complaints to dementia. Am. J. Alzheimer's Dis. Other Dement. 31, 105–114. 10.1177/1533317515592331 PMC10852868 26142292

[B46] Meyers J. Meyers K. (1995). Rey Complex Figure Test and Recognition Trial. San Antonio, TX: The Psychological Corporation.

[B47] Mueller K. D. Koscik R. L. Hermann B. P. Johnson S. C. Turkstra L. S. (2018). Declines in connected language are associated with very early mild cognitive impairment: results from the wisconsin registry for alzheimer's prevention. Front. Aging Neurosci. 9:437. 10.3389/fnagi.2017.00437 29375365 PMC5767238

[B48] Mueller K. D. Koscik R. L. LaRue A. Clark L. R. Hermann B. Johnson S. C.. (2015). Verbal fluency and early memory decline: results from the wisconsin registry for alzheimer's prevention. Arch. Clin. Neuropsychol. 30, 448–457. 10.1093/arclin/acv030 26025231 PMC4592319

[B49] Mueller K. D. Koscik R. L. Turkstra L. S. Riedeman S. K. Larue A. Clark L. R.. (2016). Connected language in late middle-aged adults at risk for alzheimer's disease HHS public access. J. Alzheimers. Dis. 54, 1539–1550. 10.3233/JAD-160252 27636838 PMC5137196

[B50] Pedregosa F. Varoquaux G. Gramfort A. Michel V. Thirion B. Grisel O. (2011). Scikit-learn: machine learning in {P}ython. J. Mach. Learn. Res. 12, 2825–2830. Available online at: https://www.jmlr.org/papers/volume12/pedregosa11a/pedregosa11a.pdf

[B51] Pistono A. Jucla M. Bézy C. Lemesle B. Le Men J. Pariente J. (2018). Discourse macrolinguistic impairment as a marker of linguistic and extralinguistic functions decline in early alzheimer's disease. Int. J. Lang. Commun. Disord. 54, 390–400. 10.1111/1460-6984.12444 30444044

[B52] Pistono A. Pariente J. Bézy C. Lemesle B. Le Men J. Jucla M. (2019). What happens when nothing happens? An investigation of pauses as a compensatory mechanism in early alzheimer's disease. Neuropsychologia 124, 133–143. 10.1016/j.neuropsychologia.2018.12.018 30593773

[B53] R Core Team. (2019). R: A Language and Environment for Statistical Computing. Vienna. Available online at: https://www.r-project.org/

[B54] Regard M. (1981). Cognitive Rigidity and Flexibility: A Neuropsychological Study. Victoria: University of Victoria.

[B55] Reisberg B. Gauthier S. (2008). Current evidence for subjective cognitive impairment (SCI) as the pre-mild cognitive impairment (MCI) stage of subsequently manifest alzheimer's disease. Int. Psychogeriatr. 20, 1–16. 10.1017/S1041610207006412 18072981

[B56] Reitan R. M. Wolfson D. (1985). The Halstead-Reitan Neuropsychological Test Battery: Therapy and Clinical Interpretation. Tucson, AZ: Neuropsychological Press.

[B57] Reppermund S. Sachdev P. S. Crawford J. Kochan N. A. Slavin M. J. Kang K.. (2011). The relationship of neuropsychological function to instrumental activities of daily living in mild cognitive impairment. Int. J. Geriatr. Psychiatry 26, 843–852. 10.1002/gps.2612 20845500

[B58] Ritchie K. Ritchie C. W. Yaffe K. Skoog I. Scarmeas N. (2015). Is late-onset alzheimer's disease really a disease of midlife? Alzheimer's Dement. 1, 122–130. 10.1016/j.trci.2015.06.004 29854932 PMC5975058

[B59] Royall D. R. Mahurin R. K. Gray K. F. (1992). Bedside assessment of executive cognitive impairment: the executive interview. J. Am. Geriatr. Soc. 40, 1221–1226. 10.1111/j.1532-5415.1992.tb03646.x 1447438

[B60] Saxton J. Lopez O. L. Ratcliff G. Dulberg C. Fried L. P. Carlson M. C.. (2004). Preclinical alzheimer disease: neuropsychological test performance 1.5 to 8 years prior to onset. Neurology 63, 2341–2347. 10.1212/01.WNL.0000147470.58328.50 15623697

[B61] Scheltens P. Blennow K. Breteler M. de Strooper B. Frisoni G. Salloway S.. (2016). Alzheimer's disease. Lancet 388, 505–517. 10.1016/S0140-6736(15)01124-1 26921134

[B62] Shao Z. Janse E. Visser K. Meyer A. S. (2014). What do verbal fluency tasks measure? Predictors of verbal fluency performance in older adults. Front. Psychol. 5:772. 10.3389/fpsyg.2014.00772 25101034 PMC4106453

[B63] Snowdon D. A. Kemper S. J. Mortimer J. A. Greiner L. H. Wekstein D. R. Markesbery W. R. (1996). Linguistic ability in early life and cognitive function and alzheimer's disease in late life: findings from the nun study. JAMA 275, 528–532. 10.1001/jama.275.7.528 8606473

[B64] Szatloczki G. Hoffmann I. Vincze V. Kalman J. Pakaski M. (2015). Speaking in alzheimer's disease, is that an early sign? Importance of changes in language abilities in alzheimer's disease. Front. Aging Neurosci. 7:195. 10.3389/fnagi.2015.00195 26539107 PMC4611852

[B65] Taler V. Monetta L. Sheppard C. Ohman A. (2020). Semantic function in mild cognitive impairment. Front. Psychol. 10:3041. 10.3389/fpsyg.2019.03041 32038404 PMC6987430

[B66] Taler V. Phillips N. A. (2008). Language performance in alzheimer's disease and mild cognitive impairment: a comparative review. J. Clin. Exp. Neuropsychol. 30, 501–556. 10.1080/13803390701550128 18569251

[B67] Tallberg I. (2005). The boston naming test in Swedish: normative data. Brain Lang. 94, 19–31. 10.1016/j.bandl.2004.11.004 15896380

[B68] Tallberg I. Ivachova E. Jones Tinghag K. Östberg P. (2008). Swedish norms for word fluency tests: FAS, animals and verbs. Scand. J. Psychol. 49, 479–485. 10.1111/j.1467-9450.2008.00653.x 18452499

[B69] Toledo C. M. Aluísio S. M. dos Santos L. B. Dozzi Brucki S. M. Sturzeneker Trés E. de Oliveira M. O.. (2018). Analysis of macrolinguistic aspects of narratives from individuals with alzheimer's disease, mild cognitive impairment, and no cognitive impairment. Alzheimer's Dement 10, 31–40. 10.1016/j.dadm.2017.08.005 29159266 PMC5675715

[B70] Unverzagt F. W. Gao S. Baiyewu O. Ogunniyi A. O. Gureje O. Perkins A.. (2001). Prevalence of cognitive impairment: data from the indianapolis study of health and aging. Neurology 57, 1655–1662. 10.1212/WNL.57.9.1655 11706107

[B71] Venneri A. Gorgoglione G. Toraci C. Nocetti L. Panzetti P. Nichelli P. (2011). Combining neuropsychological and structural neuroimaging indicators of conversion to alzheimer's disease in amnestic mild cognitive impairment. Curr. Alzheimer Res. 8, 789–797. 10.2174/156720511797633160 22175662

[B72] Wallin A. Edman Å. Blennow K. Gottfries C. Karlsson I. Regland B.. (1996). Stepwise comparative status analysis (STEP): a tool for identification of regional brain syndromes in dementia. J. Geriatr. Psychiatry Neurol. 9, 185–199. 10.1177/089198879600900406 8970012

[B73] Wallin A. Nordlund A. Jonsson M. Lind K. Edman Å. Göthlin M.. (2016). The Gothenburg MCI study: design and distribution of Alzheimer's disease and subcortical vascular disease diagnoses from baseline to 6-year follow-up. J. Cereb. Blood. Flow. Metab. 36, 114–131. 10.1038/jcbfm.2015.147 26174331 PMC4758548

[B74] Wechsler D. (2003). WAIS-III Manual (Swedish Version). New York, NY: Harcourt Assessment, Inc.

